# CAR T Cells for Solid Tumors: New Strategies for Finding, Infiltrating, and Surviving in the Tumor Microenvironment

**DOI:** 10.3389/fimmu.2019.00128

**Published:** 2019-02-05

**Authors:** Marina Martinez, Edmund Kyung Moon

**Affiliations:** Perelman School of Medicine, Abramson Cancer Center, University of Pennsylvania, Philadelphia, PA, United States

**Keywords:** chimeric antigen receptor, solid tumors, T cell, adoptive T cell immunotherapy, engineered T cells

## Abstract

Chimeric antigen receptor (CAR) T cells, T cells that have been genetically engineered to express a receptor that recognizes a specific antigen, have given rise to breakthroughs in treating hematological malignancies. However, their success in treating solid tumors has been limited. The unique challenges posed to CAR T cell therapy by solid tumors can be described in three steps: finding, entering, and surviving in the tumor. The use of dual CAR designs that recognize multiple antigens at once and local administration of CAR T cells are both strategies that have been used to overcome the hurdle of localization to the tumor. Additionally, the immunosuppressive tumor microenvironment has implications for T cell function in terms of differentiation and exhaustion, and combining CARs with checkpoint blockade or depletion of other suppressive factors in the microenvironment has shown very promising results to mitigate the phenomenon of T cell exhaustion. Finally, identifying and overcoming mechanisms associated with dysfunction in CAR T cells is of vital importance to generating CAR T cells that can proliferate and successfully eliminate tumor cells. The structure and costimulatory domains chosen for the CAR may play an important role in the overall function of CAR T cells in the TME, and “armored” CARs that secrete cytokines and third- and fourth-generation CARs with multiple costimulatory domains offer ways to enhance CAR T cell function.

## Introduction

The use of chimeric antigen receptor (CAR) T cells is gaining traction as one of the most promising advances in cancer immunotherapy. A CAR T cell is a T cell that has been genetically engineered to express an antigen-specific, non-MHC restricted receptor, composed of the single-chain variable fragment (scFv) of an antibody fused to a transmembrane domain and an intracellular signaling domain (1, 2). CARs are introduced to T cells using a plasmid or viral vector, e.g., adenovirus, retrovirus, or lentivirus, of which lentivirus has become the most common method of transducing human T cells (3). mRNA electroporated CAR T cells can also be made, with the advantage of transient CAR expression for easier evaluation of toxicity. Other nonviral vectors for integrating genes include synthetic DNA or mRNA transposon systems, termed Sleeping Beauty, in which a transposon vector can be stably integrated into the genome via a transposon plasmid with a mobilizing transposase protein (4). Importantly, the Sleeping Beauty system has been shown to be less mutagenic than retro- or lenti-viral vectors, because its genomic integration appears to be largely random, while retro- and lenti-viral vector integration is often biased toward transcriptional sites (5). The earliest first generation CARs contained only the CD3ζ signaling domain, while second generation CARs contain an additional costimulatory signaling molecule, such as 4-1BB, CD28, CD27, OX40, ICOS or RIAD, and some third- and fourth- generation CARs with two or more signaling domains have been developed as well (1, 6).

To date, the success of the CAR T cell has largely been in hematological malignancies (7, 8). A CAR targeted to the B cell antigen CD19 was first used successfully to treat chronic lymphoblastic leukemia (CLL) (9). In August 2017, the FDA approved the use of CART19 (Kymriah) to treat pediatric relapsed or refractory acute lymphoblastic leukemia (ALL) and in October of the same year, another CD19-targeting CAR (Yescarta) was approved by the FDA for adult relapsed or refractory large B cell lymphoma (10). Additionally, the European Medicines Agency (EMA) also approved the use of both these drugs in June of 2018 (11). However, despite extensive research, CAR T cell therapy for solid tumors has not been nearly as successful. Why is it more challenging to target solid tumors with CAR T cells? While there likely are numerous undiscovered reasons, the known barriers in solid tumors can be broken down into three simple categories: finding, getting into, and surviving in the tumor. This review will briefly characterize these three challenges, as well as the most recent research strategies that address them. It will focus particularly on strategies to mitigate tumor antigen heterogeneity and escape, to increase T cell trafficking and extravasation to tumor sites, and to encourage T cell proliferation in the tumor. It will address the evolving understanding of T cell activation, signaling, and the relationship between T cell memory and exhaustion phenotypes, all of which are critical for the development of more effective CAR T cells against solid tumors. Finally, research on the future of the CAR T cell, including the advent of universal CAR T cells using novel gene-editing techniques such as CRISPR/Cas9, and strategies to improve antigen-binding, optimize T cell signaling, and decrease immunogenicity, will be described.

## Finding the Tumor: Tumor Associated Antigens, Expression Level, and Susceptibility to CAR T Cells

The first major difference between solid tumors and hematological tumors is that it is more difficult to find an ideal target antigen. Unlike cancers such as ALL or CLL in which the tumor cells universally express the B-cell marker CD19, solid tumors rarely express one tumor specific antigen. For most solid tumors, it is more common to find a tumor associated antigen (TAA) where the antigen is enriched on tumors but also expressed at low levels on normal tissues (see Table 1). This is the case for many frequently targeted TAAs for solid tumors, including CEA, ERBB2, EGFR, GD2, mesothelin, MUC1, and PSMA (1, 14, 18).

**Table 1:** Common solid tumor associated target antigens, most recent CAR constructs, and the stage of testing they have reached.

**Target TAA**	**Solid tumors expressing target TAA**	**Type of CAR**	Clinical trials^*^	**Phase**
CD44v6	(Metastasized) colon cancer, soft tissue sarcoma (STS), possible marker for many metastasizing tumors (12, 13)	28ζ CAR-CIK/ HSV-TK suicide gene	Preclinical	–
CAIX (carbonic anhydrase IX)	Metastatic clear cell renal cell carcinoma (ccRCC) (14, 15)	CD4_TM_-γ	Study stopped	I/II
CEA (carcinoembryonic antigen)	Ovarian, gastrointestinal, colorectal, hepatocellular carcinoma (HCC) (16–18)	CD3ζ	NCT02959151 NCT02850536 NCT02349724 NCT03267173	I/II Ib I Early I
CD133	Ovarian, glioblastoma (GBM), HCC (17–19)	BBζ –	NCT02541370 NCT03423992	I/IIa I
c-Met (Hepatocyte growth factor receptor)	Breast (50%), melanoma, HCC (20)	BBζ mRNA c-Met/PDL-1	NCT01837602 NCT03060356 NCT03672305	Early I Early I Early I
EGFR (epidermal growth factor receptor)	NSCLC, GBM, sarcoma, malignant pleural mesothelioma (MPM) (79.2%), retinoblastoma, glioma, medulloblastoma, osteosarcoma, Ewing sarcoma (21–23)	28/BBζ α-CTLA-4/PD-1 IL12 BBζ/EGFR806/ tEGFR suicide gene	NCT03152435 NCT03182816 NCT03542799 NCT03638167 NCT03618381	I/II I/II I I I
EGFRvIII (type III variant epidermal growth factor receptor)	GBM (24–67%), glioma, colorectal, sarcoma, pancreatic (16, 24)	– tEGFR suicide gene – – BBζ+pembrolizumab –	NCT03283631 NCT02844062 NCT01454596 NCT03267173 NCT03726515 NCT03423992	I I I/II Early I I I
Epcam (epithelial cell adhesion molecule)	HCC, lung, ovarian, colorectal, breast, gastric, stomach, esophogeal, pancreatic, liver, prostate, gynecological cancers, nasopharyngeal carcinoma (16, 25)	– – 28ζ – –	NCT02915445 NCT03563326 NCT03013712 NCT02729493 NCT02725125	I I I/II I/II I/II
EphA2 (Erythropoetin producing hepatocellular carcinoma A2)	GBM, glioma (26, 27)	–	NCT03423992	I
Fetal acetylcholine receptor	Osteosarcoma, rhabdomyosarcoma (28)	CD3ζ	Preclinical	–
FRα (folate receptor alpha)	Ovarian (90%), urothelial bladder carcinoma (14)	4SCAR (4th gen)	NCT03185468	II
GD2 (Ganglioside GD2)	Neuroblastoma, melanoma, osteosarcoma (100%), rhabdomyosarcoma (13%), Ewing's sarcoma (20%), cervical (29–32)	3rd gen/inducible Caspase-9/IL-15 28ζ/OX40/iC9/VZV iC9 C7R (IL-7 receptor) 4SCAR – – – – 4SCAR/IgT	NCT03721068 NCT01953900 NCT03373097 NCT03635632 NCT02765243 NCT02919046 NCT02761915 NCT03356795 NCT03423992 NCT03356782	I I I/II I II I/II I I/II I I/II
GPC3 (Glypican-3)	HCC, squamous cell carcinoma (SCC) (17)	– BBζ/tEGFR – – – – BBζ 3rd gen – –	NCT02959151 NCT03084380 NCT02932956 NCT02905188 NCT02876978 NCT02715362 NCT03130712 NCT03198546 NCT03146234 NCT03302403	I/II I/II I I I I/II I/II I N/A N/A
GUCY2C (Guanylyl cyclase C)	Metastatic colorectal (33)	?	Preclinical	–
HER1 (human epidermal growth factor receptor 1)	Lung, prostate (1, 34)		Preclinical	–
HER2 (human epidermal growth factor receptor 2) (ERBB2)	Breast (25–30%), ovarian (25–30%), osteosarcoma (60%), GBM (80%), medulloblastoma (40%), gastric, MPM (6.3%), sarcoma, pediatric CNS (23, 24, 35–38)	BBζ/tCD19 – HER2-AdVST + oncolytic adenovirus – – 3rd gen 28ζ aE7 BBζ/tCD19 T_CM_ – –	NCT03696030 NCT02713984 NCT03740256 NCT02442297 NCT03500991 NCT03198052 NCT00902044 NCT03267173 NCT03389230 NCT03423992 NCT02792114	I I/II I I I I I Early I I I I
ICAM-1 (Intercellular adhesion molecule 1)	Thyroid (60%) (39, 40)	3rd gen	Preclinical	
IL13Rα2 (interleukin 13 receptor α2)	Glioma, GBM (41, 42)	–BBζ/tCD19	NCT03423992 NCT02208362	I I
IL11Rα (interleukin 11 receptor α)	Osteosarcoma (28)	28ζ	Preclinical	
Kras (Kirsten rat sarcoma viral oncogene homolog)	Lung adenocarcinoma (30%), pancreatic (43)	–	Preclinical	
Kras G12D	Pancreatic ductal adenocarcinoma (PDA), colorectal, lung (44)	ACT	Clinical	
L1CAM (L1-cell adhesion molecule)	Ovarian (45)	28ζ	Preclinical	
MAGE	NSCLC (MAGE-A3/6), metastatic melanoma (70% MAGE-A1-5) (46, 47)	TCR-directed therapy		
MET	MPM (67%) (48)	28ζ	Preclinical	
Mesothelin	PDA (up to 100%), MPM (85%), Ovarian (70%), lung adenocarcinoma (53%, advanced; 69%, early stage), GBM (49–52)	– ? PD-1/TCR KO αCTLA-4/PD-1 – αPD-1 PD-1 KO – αPD-1 – – BBζ 28ζ MCY-M11	NCT02930993 NCT02959151 NCT03545815 NCT03182803 NCT01583686 NCT03030001 NCT03747965 NCT03198052 NCT03615313 NCT03267173 NCT03356795 NCT02792114 NCT02414269 NCT03608618	I I/II I I/II I/II I/II I I I/II Early I I/II N/A I I
MUC1 (mucin 1)	HCC, NSCLC, pancreatic, breast, glioma, colorectal, gastric (17)	αCTLA-4/PD-1 – ± PD-1 KO T cells ± PD-1 KO T cells – – – 4SCAR-IgT –	NCT03179007 NCT02587689 NCT03706326 NCT03525782 NCT03198052 NCT03267173 NCT03356795 NCT03356782 NCT03633773	I/II I/II I/II I/II I Early I I/II I/II I/II
MUC16 ecto (mucin 16)	Ovarian (18, 53)	TCR-directed CAR	Clinical Preclinical	
NKG2D (natural killer group 2 member D)	Ewing's sarcoma, osteosarcoma, ovarian (18, 54)	NK-CAR CAR	Clinical Preclinical	
NY-ESO-1	Liposarcoma (>89%), neuroblastoma (82%), synovial sarcoma (80%), melanoma (46%), ovarian (43%), breast (46%), GBM, NSCLC (47, 55, 56)	TCR-CARACT/TCR-directed therapies	Preclinical Clinical	
PSCA (prostate stem cell antigen)	Pancreatic, prostate (57)	–	NCT03198052 NCT03267173	I Early I
WT-1 (Wilms tumor 1)	Ovarian (17)	–	Preclinical	

* *Recruiting/not yet recruiting studies listed*.

Lack of tumor antigen specificity increases the potential risk of significant on-target off-tumor toxicity. This was the case for a patient with metastatic colon cancer who received an infusion of CAR T cells targeted to the antigen HER2 (ERBB2) and died 5 days later (58). The cause of death was attributed to low levels of HER2 on the epithelial cells of the lung, which were attacked by the CARs. Another example of on-target, off-tumor toxicity was found with a high affinity anti-GD2 CAR for neuroblastoma, in which low levels of GD2 in the brain resulted in fatal encephalitis (59). These catastrophic events underscore the importance of finding a safe TAA, given the possibility that even low levels of the target antigen on normal tissues can result in significant toxicity. These acute responses also highlight that the binding affinity of a CAR is related to both safety and efficacy, and that higher affinity is not necessarily better. An *in vivo* study found that CAR T cells targeting ICAM-1, a marker associated with many solid tumors including thyroid cancer (but also expressed on many normal tissues as an adhesion marker), was safer and more effective when bearing CARs with micromolar affinity than with those with higher, nanomolar affinity (39, 40). Additionally, the authors found that the CAR with lower affinity showed less exhaustion and enhanced proliferation *in vivo*. In another approach to limiting CAR toxicity, one group interested in treating colorectal cancer created a CAR targeting GUCY2C, a receptor that is conserved in at least 95% of metastatic tumor at tenfold greater levels, but is not targeted by T cells when expressed in normal epithelial tissues because it is restricted to luminal membranes (33). The CAR was shown to be safe and effective in both immunocompetent mice with metastatic tumors and human xenograft models. Antigens that are aberrantly or overexpressed on tumors but are also expressed on normal tissues can thus be cautiously explored to serve as targets for solid tumors and their metastases.

**Figure 1:**
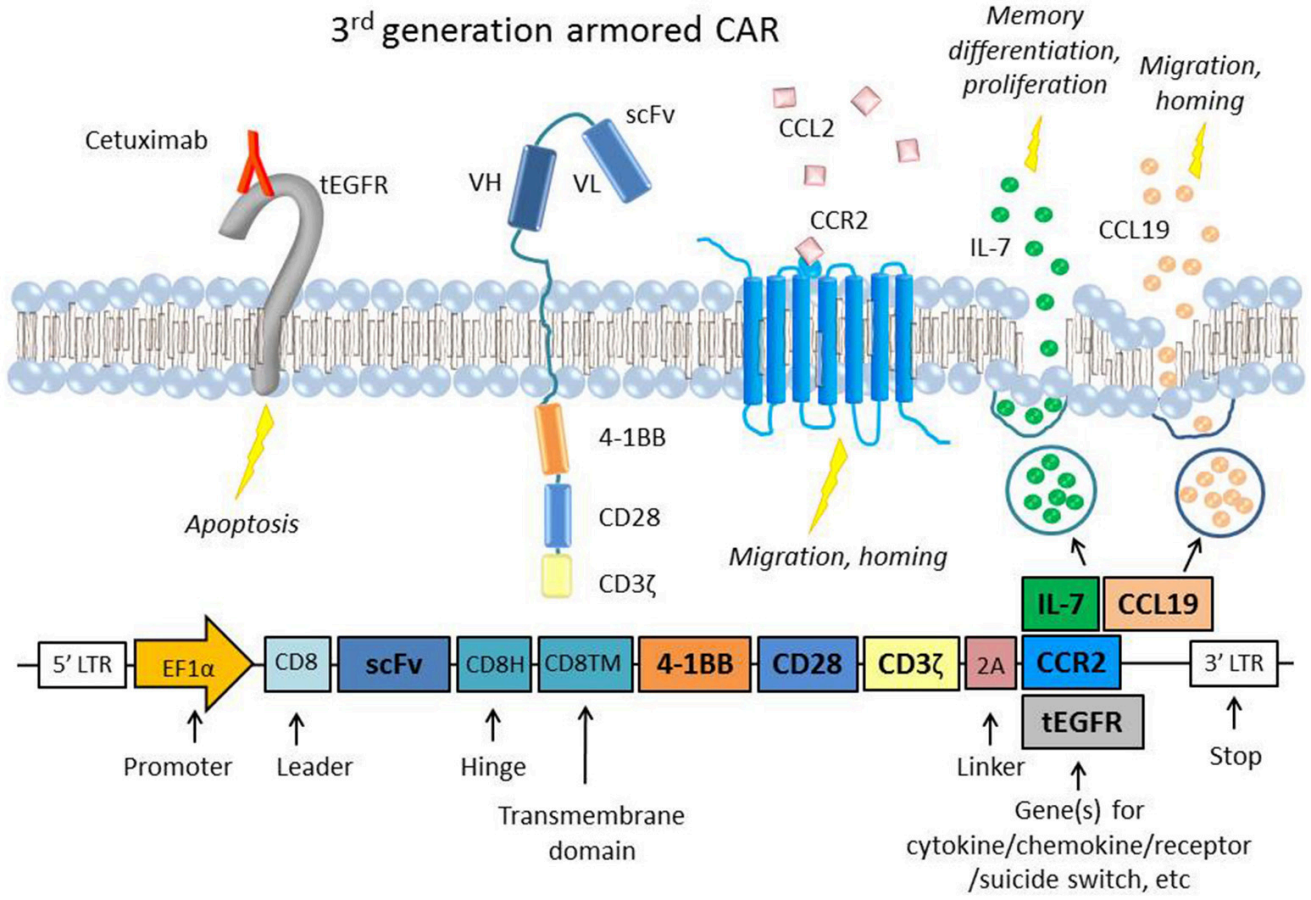
A representative figure of an armored 3rd generation CAR in a T cell and a schematic of the transgene, which includes the extracellular scFv, two intracellular costimulatory domains (4-1BB and CD28), the ζ chain, a 2A linker, and the gene of interest to be coexpressed (61, 62). Examples of “armor” added to the CAR T cell are the CCR2 receptor (63), which has been shown to increase T cell migration and homing to the tumor site (64, 65) or constitutive secretion of the cytokine IL-7 and chemokine CCL19, which are important to memory differentiation and T cell migration, respectively (66). CARs that constitutively secrete IL-12 have also been used in several studies to boost survival and cytotoxicity (67). Also depicted is an example of an inducible suicide gene, tEGFR, which consists of the truncated transmembrane and extracellular portion of the EGFR protein. When targeted by the antibody Cetuximab, the receptor triggers apoptosis in the cell, providing a safety switch to protect against potential toxicity (68). Inducible caspase 9 (iC9) and HSV-TK are other common suicide genes that have been coexpressed with CARs.

Suicide genes [reviewed by (60)] are genes coexpressed with the CAR construct that can induce cell death when activated by an agent such as a drug or antibody. Suicide genes have been integral to improving the safety of CAR T cells, particularly as they move into clinical trials. These genes include inducible caspase 9 (iC9) and truncated EGFR (tEGFR or EGFRt) (Figure 1), which can trigger antibody-mediated cell death, and herpes simplex virus thymidine kinase (HSV-TK), which disrupts DNA replication and also induces apoptosis via Fas-mediated cell death (68).

Many groups have used immunoproteomics to discover TAAs using autoantibodies against immunogenic antigens expressed by tumor cells (either on the surface or in the cytosol) (69). These antigens may be entirely unidentified proteins (neoantigens) or peptides that are mutated from the wild type (neoepitopes) (70). A few examples of TAAs identified using proteomics include the markers PSMA1, LAP3, ANXA3, and maspin, which were identified by one group as biomarkers for colon cancer (71). Other novel potentially targetable TAAs include olfactomedin 4, CD11b, and integrin alpha-2, which were found to be overexpressed in colorectal cancer with liver metastases (72). Neoantigens can also be found using DNA or RNA sequencing or whole exome screening to identify somatic mutations in tumors (73–75). A study using whole exome sequencing of melanoma samples found multiple mutated epitopes in 5 of 8 patients, as well as the presence of T cell clones reactive to 8 of the 9 neoepitopes (73). Another study used whole genome sequencing to identify somatic mutations in glioblastoma multiforme (GBM) samples, and found neoepitope-specific tumor infiltrating lymphocytes (TILs) in all five studied patients (70). Whole exome sequencing for neoantigen prediction was recently employed in a long term study of PDA patients, and the authors found that greater numbers of neoantigens, combined with greater numbers of CD8^+^ TILs, correlated with increased survival (76). Many of the neoantigens with lasting immunogenicity in long term survivors were contained within the tumor-associated MUC16 antigen; with metastatic progression, loss of MUC16 clones was seen, indicating a role for the loss of those neoantigens in tumor progression and metastasis.

Some studies have explored the use of CD40 agonists to boost T cell immunity to solid tumors (CD40 is expressed on dendritic cells and other antigen-presenting cells (APCs) and binds CD40 ligand on T cells to stimulate immune response) (77). Using CD40 agonists to augment T cell response to weakly immunogenic tumor antigens or cold tumors is particularly useful in cancers such as PDA that tend to lack mutational burden and often have no baseline immunogenicity. In a murine model of spontaneous PDA (KPC mice), combining chemotherapy with CD40 agonists showed T cell infiltration and neoantigen-specific response and tumor regression (78). The data was consistent with the hypothesis that CD40 activated pre-existing tumor-reactive TILs, showing that priming can overcome suboptimal T cell reactivity to antigen and induce an immune response with subsequent tumor control. These studies using neoepitopes show that tumors can induce secondary immune responses against previously unrecognized antigens, and that endogenous immunity to neoantigens may control tumor spread. This bears significance for adoptive T cell therapy including that which is CAR-based. Many of these methods to screen for neoepitopes rely on identifying pre-existing TCR reactivity and, thus, rely on the inherent immunogenicity of neopeptides; however, identifying neoepitopes and using CAR T cells to target them could theoretically bypass this issue because the scFv of a CAR does not rely on MHC presentation.

Solid tumors tend to display a large degree of antigen heterogeneity. Many tumors have only a subset of cells that express the target antigen. Even in the setting of a uniformly expressed TAA, there is the possibility of antigen loss or antigen escape, where the target antigen disappears from the surviving tumor (79). This has already been observed with CD19 negative relapses in leukemia post CAR19 T cell transfer, and the mechanisms are not well characterized (80). One study discovered a splice mutation that resulted in a form of CD19 that was missing the specific epitope targeted by the CD19 CAR (81, 82). In a phase I study using an EGFRvIII-specific CAR to treat GBM, a single dose of the CAR T cells resulted in downregulation of the EGFR/EGFRvIII receptor and appeared to promote T cell resistance, although administration was shown to be safe and potentially effective (83). In a glioma model, an IL13Rα2 specific CAR T cell that also had transgenic expression of IL-15 successfully killed tumor, proliferated, and produced cytokine *in vivo*; however, recurrent tumors demonstrated IL13Rα2 downregulation (84). Dual or tandem CARs, which recognize two antigens rather than one, have been created to address both antigen heterogeneity and the threat of antigen loss. Such dual CARs have entered clinical trials in hematological malignancies targeting CD19/CD20 and CD19/CD22 [(85); NCT03241940]. For solid tumors, a CAR specific for both HER2 and MUC1 had promising *in vitro* results in a breast cancer model, and a dual-target CAR specific for HER2 and IL13Rα2 showed greater success than single-target CARs in a xenograft glioma model (86, 87).

Also relevant to antigen heterogeneity is the concept of epitope spreading [reviewed by (88)], a phenomenon in which a different epitope of a previously tolerated antigen becomes targeted by T cells. In the context of CAR T cell therapy, this means that even if a tumor does not uniformly express the originally targeted antigen, lysis of some cells by CARs might release tumor-specific neoantigens or epitopes that would be processed and presented by APCs to TILs to induce a secondary immune response against the tumor. Evidence for epitope spreading has been shown in melanoma, where TILs reactive to tumor neoantigens were discovered after vaccination with melanoma antigens (MAGE) (89). Another study using a viral-based vaccine for MUC1 and IL-2 induced epitope spreading and correlated with improved survival of patients with NSCLC (90), and a case study using mRNA electroporated mesothelin CARs displayed an immune response that suggested epitope spreading in two patients with MPM and metastatic pancreatic cancer (91). In a mouse pancreatic cancer model with tumors of low mutational burden and no predicted neoepitopes, introduction of the neoantigen ovalbumin (OVA) spurred a memory immune response leading to tumor clearance and no evidence of antigen escape, while the same tumors provoked no T cell response in immune competent mice without ovalbumin (92). Further understanding and inducing epitope spreading has significant potential to bolster the effectiveness of CAR T cells, especially in tumors with high heterogeneity, low mutational burden, and evidence of antigen escape.

For traditional CAR T cells, the target antigen must be expressed on the cell surface in order to engage with a T cell. However, only about 1% of total cellular proteins are actually expressed on the cell surface, meaning that a huge number of potential tumor target antigens are not available to a CAR T cell (62). Recently, to open the doors to targeting intracellular antigens with CAR T cells, Patel et al. (93) showed success in an *in vivo* myeloma study with a CAR/TCR hybrid that recognized the antigen NY-ESO-1 in the context of HLA-A2. These TCR-CARs were shown to effectively bind an HLA-A2^+^ T cell artificially engineered to express NY-ESO-1. TCR-CARs that recognize antigen in combination with MHC can thus recognize both extra- and intra-cellular antigens in the way that wild-type or modified TCRs can. Walseng et al. (62) also created a TCR-CAR composed of a soluble TCR directed against either the melanoma-associated antigen MART1 or TGFβR2 (a neoantigen peptide) joined to a CAR signaling component. The result was a versatile receptor that bound antigen in an MHC-I restricted manner, but with signaling and killing similar to that of a CAR. They demonstrated that this construct could be transduced not only into T cells but also into a NK cell, with successful *in vitro* killing.

## Tumor Infiltration

Even when a target antigen for a solid tumor is identified, a CAR T cell must be able to reach the tumor site. In hematological cancers, circulating CAR T cells in the bloodstream have already reached their destination. In solid tumors, there are multiple barriers that a CAR T cell must surmount in order to reach the tumor site [for full reviews of the tumor microenvironment, see (94, 95)]. Chemokine-receptor mismatch can prevent migrating lymphocytes from following a chemotactic gradient. Surface markers like selectins on endothelial cells that bind circulating lymphocytes and induce signaling cascades for subsequent extravasation into sites of inflammation are necessary, as are the corresponding receptors on T cells. Additionally, physical barriers such as cancer associated fibroblasts (CAFs) and abnormal vasculature at the tumor site can block T cell entry (95).

The presence of blood vessels known as high endothelial venules (HEVs) are hypothesized to be critical for T cell infiltration and have been associated with tumor regression in cancers such as melanoma. However, these blood vessels are distorted and immature in many solid tumors, particularly at the core of the tumor where the fewest TILs are found (96). Anti-angiogenic therapy targeting VEGF, CD276, or endothelin B receptor has been shown to normalize tumor vasculature and could be used in combination with targeted therapy like CAR T cells to increase tumor infiltration (97, 98). Notably, one study that performed qPCR on melanoma lesions observed that high HEV density positively correlated with the number of genes encoding for chemokines known to recruit TILs, including CCL2, CCL5, CXCL9-13, CCL19, and CCL21 (99). In colorectal cancer, expression of CXCL9, 10 and 11 were positively correlated with the presence of CD8^+^ and CD4^+^ TILs and with post-operative survival (100).

Given the importance of chemokines in lymphocyte migration and homing, varying methods have been used to deliver chemokines intratumorally to attract TILs. One study employed a vaccinia virus to deliver the chemokine CXCL11 intratumorally in a subcutaneous mouse model of MPM and observed significantly increased levels of T cell infiltration and anti-tumor efficacy after intravenous mesothelin-directed CAR T cell injection (101). The same group also developed a CXCL11/mesothelin CAR that increased intratumoral levels of CXCL11 but did not improve anti-tumor activity. The investigators hypothesized that this was due to chronic chemokine secretion inducing hypofunction in the T cells, and/or the anti-angiogenic effects that CXCL11 can exert on its surroundings. However, another study that engineered “armored” mesothelin CAR T cells that constitutively expressed both the cytokine IL-7 and the chemokine CCL19 showed complete tumor regression and prolonged survival in a solid tumor mouse model (Figure 1) (66). The study also showed that lymphodepletion before CAR T cell injection decreased efficacy, suggesting that IL-7 and CCL19 recruited endogenous anti-tumor TILs as well. CAR T cells have also been transduced to express chemokine receptors with beneficial results, as in the case of lentivirally engineered mesothelin CAR/CCR2 T cells that displayed greater than 12-fold increased homing and tumor regression in subcutaneous human MPM tumors and a GD2/CCR2b CAR T cells that showed greater than 10-fold increased homing in neuroblastoma tumors *in vivo* (Figure 1) (64, 65).

Another promising approach to augment CAR T cell infiltration into tumor sites is the development of a CAR targeting FAP (fibroblast activation protein), which is expressed on multiple types of stromal cells that are associated with nearly all epithelial tumors (102). FAP has been shown to play a role in epithelial-to-mesenchymal transition (EMT) in pancreatic ductal adenocarcinomas (PDA) among other tumor types (103). In one study, where human MPM tumor samples and fibroblast samples were shown to be positive for FAP by immunohistochemistry, FAP CAR T cells efficiently killed MPM cells *in vitro*. The same CAR T cells inhibited tumor growth and lengthened the survival of immunodeficient mice with intraperitoneal (IP) tumor xenografts (104). However, another study showed little efficacy of a FAP CAR in a syngeneic mouse model using multiple tumor types and observed lethal toxicity, which was attributed to FAP expression on bone marrow-derived stem cells (BMSCs) (105). The authors reported that this may have been due to the use of mouse tumor lines with limited FAP expression, while robust FAP staining by IHC was observed in multiple human tumor samples, indicating that human tumor cell lines may have been better targets for the study.

One method of entirely circumventing the hurdle of suboptimal T cell homing (and also potentially avoiding on-target off-tumor toxicity) is regional/local CAR T cell administration, which has already been tested in patients with solid tumors with varying degrees of success. One phase 0 study that enrolled patients with metastatic breast cancer demonstrated that intratumoral administration of mRNA c-Met CAR T cells was safe and resulted in tumor cell death, and showed other signs of anti-tumor inflammation including macrophage recruitment (20). Recently, in a study using a xenograft mouse model of human breast cancer metastatic to the brain, intracranial and intratumoral administration of HER2-specific CAR T cells showed improved antitumor activity compared with intravenous delivery, with complete tumor eradication and 100% survival even after tumor rechallenge (106). Another study showed that regional delivery of a HER2-BBζ CAR T cell cleared medulloblastomas in NSG mice and required a significantly lower dose than intravenous delivery (107). The same CAR in nonhuman primates with HER2 positive medulloblastomas showed no toxicity after intraventricular delivery. A mouse study using a CEA CAR for peritoneal carcinomatosis (colorectal cancer metastasized to the peritoneal cavity) showed that regional intraperitoneal (IP) delivery resulted in better antitumor response than intravenous delivery, even after tumor rechallenge and at distal tumor sites (108). Finally, a study of intracavitary administration of pan-ErbB/IL-4 CAR T cells targeting patient derived MPM xenografts in SCID mice showed tumor regression or cure in all mice (23).

## TIL Survival in the Tumor Microenvironment

**Figure 2:**
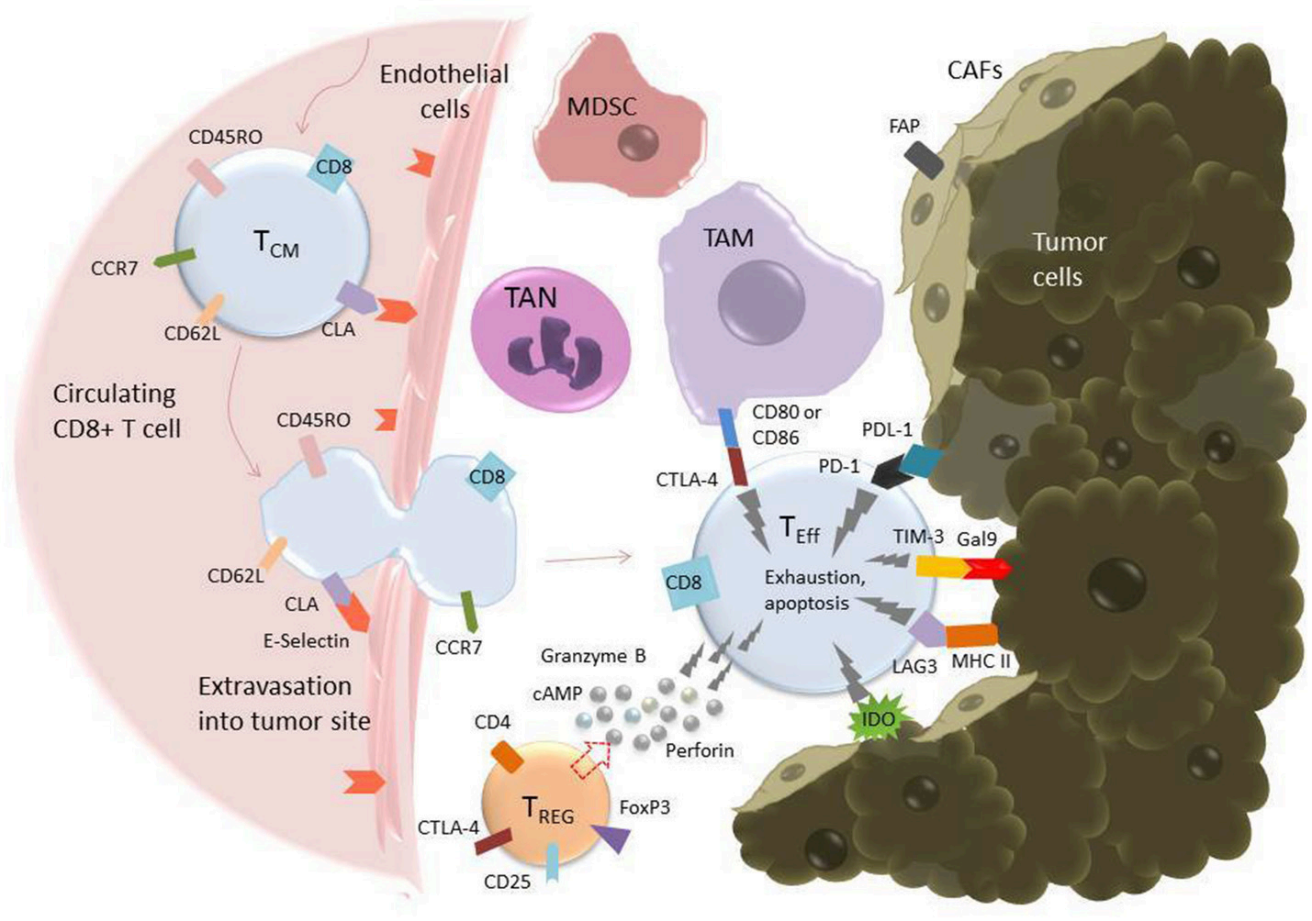
T cell extravasation into the TME and subsequent exhaustion mediated by inhibitory ligands on tumor and tumor-associated cells. Endothelial cells experiencing inflammation express adhesion molecules including selectins, vascular cell adhesion molecule-1 (VCAM-1), and intercellular adhesion molecule (ICAM-1). P- and E-Selectins (the latter shown in the figure) bind cutaneous lymphocyte antigen (CLA), a specially glycosylated form of P-selectin glycoprotein ligand 1 (PSGL-1) that is expressed on activated T cells (114). VCAM-1 binds very late antigen-1 (VLA-4) and ICAM-1 binds lymphocyte function-associated antigen-1 (LFA-1) (115). Upon binding endothelial cell ligands, T cells undergo tethering and rolling before adhering to the endothelium and transmigrating through it as shown. Once in the tumor microenvironment, T cells are in an environment full of tumor-associated, immunosuppressive cells including tumor-associated macrophages (TAMs), tumor-associated neutrophils (TANs), myeloid-derived suppressor cells (MDSCs), T-regulatory cells (Tregs), and cancer-associated fibroblasts (CAFs) (95). These cells express inhibitory molecules, including CD80/CD86, which bind the inhibitory receptor CTLA-4 (pictured), and secrete soluble factors that suppress or cause apoptosis in T cells. CAFs also serve as a physical barrier between T cell and tumor cell. Additionally, tumor cells themselves express ligands such as Gal9 and PDL-1, which bind to the T cell inhibitory receptors TIM-3 and PD-1, respectively. All these factors serve to promote an “exhausted” phenotype in the T cell, characterized by upregulation of inhibitory receptors such as PD-1, TIM-3, TIGIT, and LAG-3, loss of CCR7, CD62L, and CD45R0, loss of cytotoxicity, and apoptosis (111).

**Figure 3:**
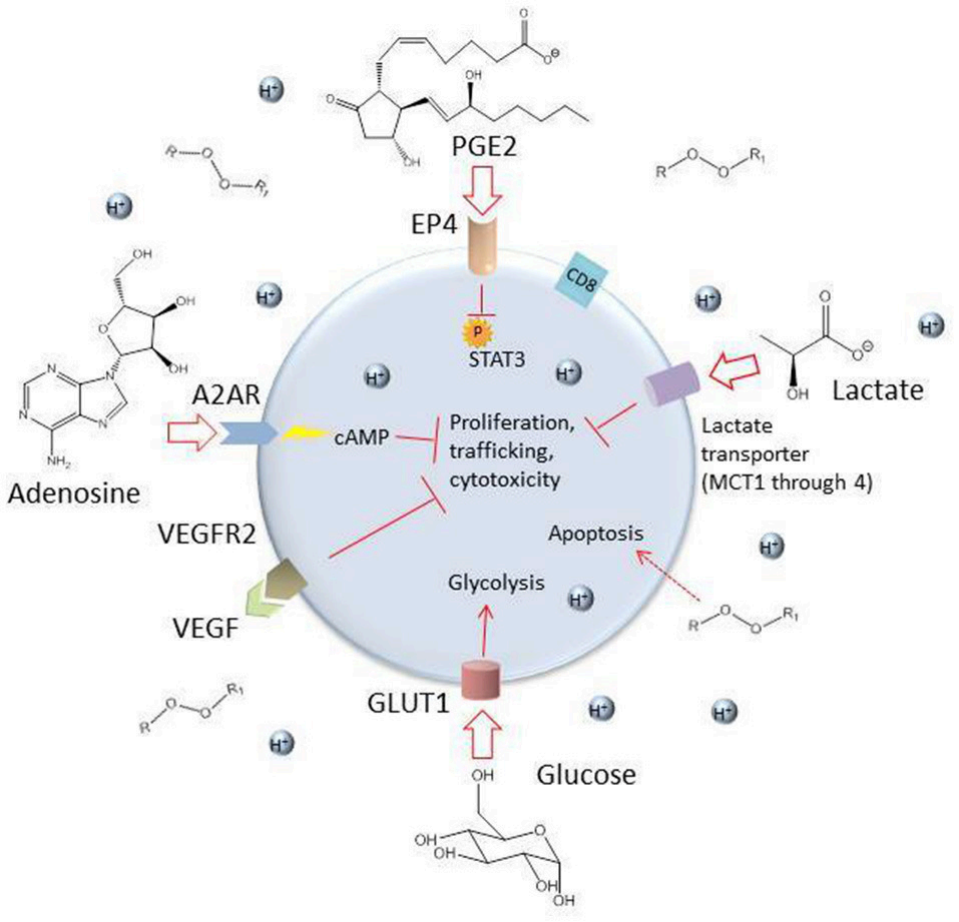
Some inhibitory soluble factors and molecules secreted by tumor cells and tumor-associated cells such as MDSCs, TAMs, TANs, CAFs, and Tregs. High levels of lactate and an acidic environment are generated because of the tumor cells' preferential use of glycolysis, which impairs T cell function (111). The hypoxic environment also limits oxidative phosphorylation, a metabolic requirement for central memory T cells. High levels of reactive oxygen species (ROS) are generated by tumor cells and by induced mitochondrial dysfunction in T cells, which can be toxic to the cell. The soluble factors VEGF, TGFβ, indoleamine 2,3-dioxygenase (IDO), prostaglandin E2 (PGE2), and adenosine are secreted by tumor and tumor-associated cells and can have damaging effects on T cells (109, 117). Adenosine enters the T cell through the receptor A2AR and stimulates production of cyclic AMP, which inhibits T cell proliferation, trafficking and cytotoxicity (118). PGE2 enters through the receptor EP4 and inhibits phosphorylation of STAT3, dampening proliferation, development of favorable memory phenotype, and cytotoxic function in T cells (119). Competition with glycolytic tumor cells for glucose results in downregulation of the glucose receptor GLUT1 because of decreased AKT/mTOR signaling and consequently, the T cell's metabolic capacities are further diminished (120).

Once a CAR T cell finds its way into the tumor, the battle is far from over. The tumor microenvironment (TME) has been extensively characterized as hostile for T cells [see (95, 109), and (110) for reviews of the tumor microenvironment and the different cell types it comprises]. The glycolytic metabolism of tumor cells renders the environment hypoxic, acidic, low in nutrients, and prone to oxidative stress (1, 109). In an inflammatory environment, tumors cells often upregulate ligands such as programmed cell death ligand 1 (PD-L1) and Galectin-9 that bind to inhibitory receptors on T cells (see Table 2). The tumor microenvironment also relies on stromal cells like cancer associated fibroblasts (CAFs) and suppressive immune cells, including myeloid-derived suppressor cells (MDSCs), tumor associated macrophages (TAMs), tumor associated neutrophils (TANs), mast cells, and regulatory T cells (Tregs) (Figure 2) (95). These cells and tumor cells secrete soluble factors like vascular endothelial growth factor (VEGF) and transforming growth factor β (TGFβ), which contribute to abnormal tumor vasculature, promote anti-inflammatory polarization of TAMs and other immune cells, and are implicated in EMT (116). They also produce reactive oxygen species (ROS) and molecules like lactate, indoleamine 2,3-dioxygenase (IDO), prostaglandin E2 (PGE2), soluble Fas, and adenosine, which contribute to the suppression of the T cell immune response (Figure 3) (117, 121).

**Table 2:** Some inhibitory receptors and their known ligands [from Wherry et al. (111), unless cited in table].

**Inhibitory receptor**	**Full name**	**Ligand(s)**
A2AR	Adenosine 2A receptor	Adenosine
CTLA-4	Cytotoxic T lymphocyte antigen-4	CD80, CD86
CD160	Cluster of differentiation 160	MHC Class I, herpesvirus entry mediator (HVEM) (112)
LAG-3	Lymphocyte activation gene 3	MHC Class II
PD-1	Programmed cell death 1	Programmed cell death ligand 1 (PD-L1), PD-L2
TIM-3	T cell immunoglobulin-3	Galectin-9 (Gal9), phosphatidylserine (PtdSer), high mobility group protein B1 (HMGB1), Ceacam-1 (113)
TIGIT	T cell immunoglobulin and ITIM domain	PVR (CD155) >> PVRL2 (CD112), PVRL3 (113)

When they are activated, effector T cells generally switch from oxidative phosphorylation (oxphos) to glycolysis to facilitate faster proliferation, while memory T cells and Tregs rely on oxphos and fatty acid oxidation (FAO) when activated [for a full review on T cell activation and metabolism see (122)]. However, both of these metabolic resources are limited in the TME because glucose is depleted by tumor cells, leaving glycolytic T cells nutrient-deprived. The lack of glucose results in lowered AKT/mTOR signaling, downregulation of the glucose receptor Glut1, and reduced capacity for glycolysis (120). Low oxygen concentrations in the TME limit oxphos as well. Overall, this results in significant depletion of both major sources of T cell nutrients. In a study of resected tumor tissue from 54 patients with clear cell renal carcinoma (ccRCC), CD8^+^ TILs showed very low levels of activation and proliferation, and although T cell Glut1 remained expressed, TILs did not uptake glucose (123). The study also observed that mitochondria (crucial for T cell activation), undergo remodeling during glycolysis, lose function and release detrimentally high levels of ROS. This comprehensive study is one of many to show the effects of TME hypoglycemia on suppressing T cell activation via glycolytic and mitochondrial pathways.

The hypoxic conditions in the TME provide particular challenges for memory T cells, the metabolisms of which rely heavily on oxygen. Some studies have recently begun to identify and test modifications to CAR T cells to improve their function in low-oxygen conditions. Kawalekar et al. (6) found that BBζ CAR T cells had increased mitochondrial spare respiratory capacity (SRC) compared with 28ζ CAR T cells, resulting in greater metabolic efficiency even in nutrient-poor, oxygen depleted conditions such as the TME. Because the BBζ costimulatory has been implicated in promoting memory-associated metabolic pathways such as fatty acid oxidation as well as increased persistence (further discussed under “Intracellular signaling pathways of the CAR” below), the increased SRC observed in these T cells was hypothesized to aid their survival in low-oxygen conditions.

One approach designed to protect T cells from the oxidative stress inflicted by ROS in the TME was the design of a CAR T cell coexpressing catalase, an enzyme that reduces hydrogen peroxide to water and oxygen (124). The authors tested both CEA and HER2 CAR T cells *in vitro* and found that CAR-CAT displayed a reduced oxidative state and improved proliferation and cytotoxicity compared with CAR alone. Another study harnessed the hypoxia associated with the TME to develop a CAR coexpressing the oxygen-sensitive domains of HIF1α, a transcription factor that is stabilized in response to hypoxia (125). *In vitro*, the strategy enabled very low CAR expression at normal oxygen levels, but highly increased levels of CAR expression together with HIF1α in hypoxic conditions. While this approach does not address the detrimental effects of low oxygen or ROS in the TME, it does provide proof of concept for a novel type of safety switch that uses the hypoxic TME to a therapeutic advantage.

T cell exhaustion [reviewed by (111)] is characterized by chronic antigen exposure that spurs loss of effector and memory phenotypes, inability to produce cytokines like IFNγ, TNFα, and IL-2, and upregulates expression of inhibitory receptors (IRs) that further shut down effector functions upon binding to inhibitory ligands or soluble factors in the TME (Table 2) (109).

### Checkpoint Blockade

One of the most popular and successful strategies to combat T cell exhaustion is the use of checkpoint inhibitors, in which either an IR or its ligand is blocked with an antibody. Drugs targeting PD-L1 (atezolizumab), PD-1 (pembrolizumab, nivolumab), and CTLA-4 (ipilimumab) have been used independently and in combination with CAR T cell therapy with success in many patients [reviewed in (126)]. Currently, atezolizumab and pembrolizumab are used to treat metastatic NSCLC and are being actively studied for use in other solid tumors as well. Pembrolizumab was recently approved by the FDA for first line use in combination with chemotherapy in lung cancer (127, 128). Nivolumab has shown significant responses in a phase I/II trial with HCC, among others (17). Ipilimumab was shown to lengthen the survival of metastatic melanoma patients in a phase III study from 2010, and it has shown promising results in mouse mesothelioma models as well as in many other preclinical studies (129, 130). Ipilimumab has also been used in combination with VEGF inhibitors to treat metastatic melanoma in phase I trials. In one study, anti-CTLA-4 therapy combined with anti-VEGF antibodies resulted in an increase in anti-tumor response resistant to the immunosuppressive effects of the ligand galectin-1 (131). Another study by the same authors showed ipilimumab and anti-CTLA-4 therapy resulted in humoral immunity to galectin-3, which is also a suppressive tumor ligand (132).

Preclinically, combining CARs with checkpoint blockade antibodies has shown promising results. CAR T cells have also been engineered to secrete checkpoint inhibitor antibodies themselves. Anti CAIX CAR T cells engineered to secrete anti-PD-L1 antibodies showed significantly improved activity compared to standard CAR T cells, with increased cytokine production and immune cell recruitment as well as significantly reduced tumor size in a human ccRCC mouse model (14). In another study, a CAR19 T cell designed to constitutively secrete anti-PD-1 also showed enhanced anti-tumor activity in a CD19^+^ lung cancer xenograft model, with increased T cell proliferation and cytotoxicity, and prolonged survival (133). A similar study also used MUC16-ecto targeting CARs secreting anti-PD-1 scFvs in syngeneic and xenograft mouse models of PD-L1^+^metastatic ovarian cancers, and showed superiority over CAR T cells plus PD-1 checkpoint inhibitors (134). Along a similar line of thinking, an anti-PD-L1 CAR has shown *in vitro* cytotoxicity (135); it has yet to be seen whether these CARs might be successful *in vivo*, either alone or as adjuvant therapy. CAR T cells engineered to secrete PD-1, CTLA-4, or PD-L1 antibodies have gone to clinical trials for MUC1, EGFR, EGFRvIII, and mesothelin expressing cancers (136).

Dominant negative genes for IRs have also been successfully introduced to CAR T cells in many preclinical studies, as in the case of a mesothelin CAR T cell (with either a CD28 or 4-1BB costimulatory domain) overexpressing dominant negative PD-1 (137). The authors observed tumor clearance with the dominant negative PD-1 CARs, while repeated doses of PD-1 blocking antibody in combination with either the mesothelin-28ζ or mesothelin-BBζ CAR was able to prevent growth but not eradicate the tumor. These results show that a genetic built-in resistance to checkpoint inhibition has advantages over a blocking antibody that must be repeatedly administered and may cause resistance. Recently, CRISPR/Cas9 technology has been used to knock out the gene for the IR itself, which has been done for both PD-1 and LAG-3 in CD19-BBζ CAR T cells. In both cases, tumors were eradicated in mouse xenograft models using the IR knockout CAR T cells (138, 139). This approach has recently been translated to solid tumors as well: CRISPR/Cas9 was used to knock out PD-1 in T cells while simultaneously transducing them with a CD133-specific CAR, and the resulting PD-1 KO CARs showed improved tumor control in a mouse glioma model compared with control CD133 CARs (140).

With the optimization of gene editing methods, CRISPR/Cas9 edited CARs are already moving into clinical trials: a PD-1 knockout CD19 CAR has is being studied in a phase I clinical trial (NCT03298828).

Switch receptors are designed to mitigate the effects of inhibitory ligands on T cell function while simultaneously enhancing T cell activity. In a switch receptor, the ligand-binding external IR domain is fused to the cytoplasmic signaling domain of an activating molecule. For example, a PD-1/CD28 switch receptor was engineered into mesothelin-BBζ or PSCA-BBζ CAR T cells, and both switch-receptor CARs performed significantly better than wild type CARs at eradicating tumor in xenograft NSG mouse models (141). In a breast cancer model, the investigators engineered a MUC1 CAR that coexpressed a cytokine switch receptor (4/7ICR) with an IL-4 receptor extracellular domain fused to an IL-7 intracellular signaling domain (142). The 4/7ICR MUC1 CARs proliferated, suppressed tumor growth *in vivo* and did not show markers of exhaustion, while MUC1 CARs without the switch receptor did. PSCA CAR T cells that also contained a 4/7ICR switch receptor proliferated and killed better in the presence of IL-4 and showed significantly improved tumor reduction compared to T cells with the CAR alone in NSG mice with subcutaneous pancreatic cancer (57).

Similar to switch receptors are bispecific T cell engagers (BiTEs), which also subvert suppressive signals from the TME by binding both a tumor ligand and a T cell marker (i.e., CD3). Recently, a humanized EGFRvIII-specific scFv linked to an anti-CD3 scFv showed significant control of glioma xenografts and prolonged survival of mice (143). Potentially, the use of bispecific antibodies in conjunction with CAR T cells could play a role in recruiting TILs and in deterring immunosuppressive signals. Another strategy is to have a fusion protein bind not to the T cell but to the tumor itself. Recently, a PD-L1/TGFβR2 fusion protein was developed for use with TGFβ expressing urothelial carcinoma (144). In this study, a PD-L1 antibody fused to a TGFβ receptor was able to accomplish both blockade of PD-L1 on the tumor and binding of TGFβ to attenuate its presence in the TME. Excitingly, the authors also observed a greater presence of chemokines like CXCL11 in the tumor as well as antigen-specific killing by T cells. With the right target tumor ligands, BiTEs could be a promising strategy to augment the function of CAR T cells in solid tumors.

Understanding the metabolism and transcriptional profiles of exhausted or exhausting TILs has significant impacts on the success of therapies like checkpoint blockade and could lead to the production of more functional CAR T cells via metabolic reprogramming. In a mouse melanoma model, one group showed that promoting fatty acid catabolism in vaccine-induced CD8^+^ TILs using a PPARα agonist combined with anti PD-1 therapy significantly improved anti-tumor activity (145). Another group showed that CTLA-4, PD-1, and PD-L1 blockade increased glucose concentrations in the TME to favor glycolysis in T cells, improving their function in a mouse sarcoma model (120). These studies provide clues into the roles of ligands like PD-L1 on tumor metabolism, in addition to their known inhibitory effects on T cell IRs.

Despite the success of PD-1 therapy in treating NSCLC and melanoma, as well as its use in multiple other clinical trials, it is inadequate to characterize markers like PD-1 as solely inhibitory. Much of what we know about PD-1 in the context of exhaustion comes from chronic viral infection models, and it has become clear that PD-1 can in fact be a marker of activation and positive prognosis if expressed on certain subsets of T cells in cancer, while some subsets of exhausted T cells may have low PD-1 expression (111, 141). In the same study by Zhang et al. (145) that found a synergistic effect of PD-1 blockade with fatty acid catabolism in a melanoma model, hypoxia-driven hypofunction in CD8^+^ TILs was accompanied by lower PD-1 expression (but increased LAG-3 expression), and increased PD-1 signaling was hypothesized to be associated with metabolic reprogramming from glycolysis to FAO in low glucose environments like the TME. Another study suggested that PD-1 was in fact not linked to exhaustion, and that prolonged antigen exposure alone could cause T cells to become exhausted (146). In a recent analysis of varied NSCLC patient samples, a population of CD8^+^ TILs with high PD-1 expression did not appear exhausted, and genes involved in cycling and proliferation such as Ki-67, as well as genes involved in trafficking and metabolism, were upregulated (147). The PD-1 high TILs also showed greater glucose, lipid and fatty acid uptake than patient TILs with lower PD-1 expression. This data challenges the understanding of PD-1 being solely an inhibitory receptor and sheds new light on Zhang et al.'s observation that an increase in PD-1 expression results in lower capacity for glycolysis. Further showing a role for PD-1 outside of exhaustion, a study comparing CD4^+^ TILs in 34 patients with metastatic melanoma, grouped into young vs. old, showed that the younger patients had a greater percentage of memory T cells that expressed PD-1, Ki-67, and HLA-DR (another activation marker), compared with age matched controls; these memory and activation phenotypes were less distinct in older patients (148). However, supporting the hypothesis of PD-1 as a marker of hypofunction, but not necessarily of terminal exhaustion, one study demonstrated that mesothelin/BBζ T cells that had high PD-1 expression and a hypofunctional phenotype in an vivo human mesothelioma model recovered the ability to produce cytokines and had lower PD-1 expression after 24 h out of the tumor (126). These data show that PD-1 can have highly variable functions which likely depend on T cell phenotype, metabolism, tumor type, and other factors in the TME, and also helps explain why only a fraction of patients respond to PD-1 blockade even when their tumors have high PD-L1 expression.

### Transcription Factors

Transcription factors such as T-box transcription factor TBX21 (T-bet) and Eomesodermin (Eomes) are involved in determining T cell fate and their discovery has led to further insight into the mechanisms of T cell exhaustion. T cells high in Eomes and PD-1 have been shown to be terminally exhausted, while those with high T-bet and medium PD-1 levels appear to retain proliferative potential despite displaying other classically defined features of exhaustion (111). Hypoglycemia and hypoxia in the TME have been shown to decrease T-bet expression in TILs that also lose effector functions (145). As an example of Eomes' role in CAR T cell exhaustion, a case study with a patient with refractory diffuse large B cell lymphoma (DLBCL) who received CAR19 T cell therapy combined with PD-1 blockade showed decreased Eomes as well as decreased PD-1 levels in peripheral blood CAR T cells (149). The patient had a clinically significant response to the treatment, indicating that PD-1 blockade improved the efficacy of the CAR T cells.

Other transcription factors and signaling cascades have been investigated as biomarkers to predict T cell function and patient prognosis after adoptive transfer of CAR T cells. A study using IL-18/CEA CAR T cells to treat immunocompetent mice with advanced pancreatic carcinoma showed that constitutive secretion of the proinflammatory cytokine IL-18 resulted in CARs with high T-bet in conjunction with low levels of the transcription factor FOXO1 and showed improved antitumor efficacy (150). In an analysis of CAR19 T cells derived from patients with CLL, the circulating CARs from complete responders had upregulated genes associated with memory differentiation status, including IL-6 and STAT3, and were observed to lose function upon IL-6/STAT3 blockade (119). Nonresponders, on the other hand, had upregulated genes associated with more of an effector phenotype as well as glycolysis, exhaustion and cell death by apoptosis. Finally, the paper showed that CD8^+^ cells that were CD27 positive and PD-1 negative were positively predictive of response to CAR19 T cell therapy. Investigating the role of these biomarkers in CAR T cell response for solid tumors and whether they have an impact on patient survival may elucidate the transcriptional profiles of functional CARs.

### Differentiation and Memory

Naive, central memory and effector memory, and terminally differentiated effector T cells all have distinct markers of differentiation. Differential expression of CCR7, CD62L, CD25, CD45RA, CD45R0, CD95, and IL-7Rα, among others, can identify subsets of T cells (151, 152). Tregs, CD4^+^ T cells that express CD25, CTLA-4, and FOXP3, are another distinct subset that inhibits T cell effector function; studies have shown that with CAR T cell therapy, Treg presence lowers CAR antitumor activity. Checkpoint blockade targeting CTLA-4 may be one way to address this problem. Other surface markers like IL-2Ra and KLRG1 have been shown to be associated with effector-like phenotypes, while IL-7Rα and the chemokine receptor CXCR3 are associated with memory-like cells (153). Cytokines such as IL-2, IL-12, IL-27, and IFNγ are also traced to effector-differentiated T cells, while IL-10, IL-21, IL-7, IL-15, and TGFβ are associated with a memory phenotype. Genes such as T-bet, Id2, Blimp-1, Batf and Stat4 have been associated with effector phenotypes, while Id3, Bcl-6, Tcf-7, Stat3, Foxo1, and Eomes are all proposed to be upregulated in memory-like T cells (150, 153).

Gattinoni et al. (154) describe a human stem cell memory T cell (TSCM) population that expresses both the classical markers of naive cells as well as certain memory cell markers including CD95 and IL-2Rβ, which the authors determined to be crucial identifiers of TSCM. These cells were found in 2–3% of circulating blood lymphocytes of healthy donors, and could also be induced from naive T cells by culturing them in the presence of a glycogen synthase kinase 3β (GSK3β) inhibitor. (Inhibition of GSK3β has been described to stabilize β-catenin and halt differentiation to effector T cells while promoting memory characteristics). These TSCM cells demonstrated increased proliferation in response to the cytokines IL-7 and IL-15 compared with effector memory T cells, while still retaining their phenotype.

The memory phenotype has consistently been shown to favor T cell survival, proliferation, and prolonged presence of TILs at tumor sites. However, only a few studies focusing on T cell differentiation and memory up to this point have been done in solid tumor models. Still, valuable lessons can be learned from models of ACT, GVHD, or hematological malignancies. For example, a study of ACT in both NSG mice and nonhuman primates showed that purified TCM cells persist better than standard T cells, and form stable memory pools (155). Cieri et al. (156) published an *in vitro* culture protocol for inducing a stem cell memory-like phenotype in T cells using IL-7 and IL-15 during CD3/CD28 bead activation of naive precursor cells. When transduced with a transgenic TCR, these T cells displayed memory-associated qualities including proliferation, cytokine production, and expression of CD45R0 upon exposure to target antigen, while retaining markers indicative of naive T cells including CD45RA and CD62L. These T cells were very similar to those described by Gattinoni et al. (154) above, except for the expression of CD45R0. The authors found enhanced proliferation in these cells compared with cells expressing a central memory or effector memory phenotype, and this translated to increased expansion and cytotoxicity compared with other subsets of T cells in a mouse GVHD model. The results of these experiments suggest that, as stated by the authors, using naive cells for ACT may significantly improve clinical outcomes. Similarly, using IL-7/IL-15 with naive T cells during transduction and culture conditions to produce more TSCM CAR T cells is a promising strategy for the creation of CAR T cells that both kill and proliferate better in the body. In one such study for solid tumors, T cells bearing a third-generation GD2 CAR (signaling domains for CD28 and OX40), as well as an inducible caspase 9 suicide gene, were activated with CD3/CD28 stimulation along with a variety of cytokines, with the hope of identifying conditions to promote a memory phenotype (157). Addition of IL-7 and IL-15 led to the greatest antigen-specific cytotoxicity *in vitro* along with the highest percentage of stem cell memory and central memory subsets as identified by CD45RA, CCR7, and CD95. The authors predicted better proliferation, survival, and antitumor activity of GD2-CD28-OX40 CAR T cells cultured in IL-7/IL-15.

Clinically, work has been done to determine biomarkers associated with memory that are predictive of response as well as to actually manufacture CAR T cells with optimized differentiation status for infusion into patients. In a study examining T cell memory in ovarian carcinoma patients, it was found that increased presence of CD8^+^ effector memory cells, as well as the chemokine CXCL9, was significantly associated with long-term survival (158). The authors also implicated the signaling proteins STAT5B, PLCγ1, and NFATc2 as being relevant to survival, with lower levels of these signals correlating with hypofunctional T cells and shorter survival times in patients.

In the study by Fraietta et al. (119) discussed above, the authors looked at both the original unmodified T cells from CLL patients and the corresponding CAR transduced infusion products. All sets of T cells from responding patients showed markers associated with early memory, non-exhausted T cells. As glycolysis is a hallmark of effector/exhausted T cell metabolism, and the T cells of nonresponders displayed upregulated genes for exhaustion and glycolysis, the authors used a glycolysis inhibitor while manufacturing CAR T cells and observed increased numbers of memory CAR T cells along with enhanced proliferation upon exposure to antigen. Blocking glycolysis is another approach that could be used in solid tumor-targeting CAR T cells to push formation of memory T cells during activation and transduction, particularly given (119) evidence that the initial differentiation status of a patient's apheresed T cells may significantly affect the efficacy and persistence of the infusion product. Memory CAR T cells have been manufactured for clinical use already in hematopoetic malignancies such as leukemia; Wang et al. (155) used a protocol using magnetic separation to select CD8^+^ CD45RA^+^ CD62L^+^ TCM cells to transduce with a CD19 CAR while culturing with IL-2 and IL-15, and several variations of these CARs (which have CD28 costimulatory domains) are being tested in a phase I study. Also for leukemia, a GMP protocol for manufacturing CAR T cells highly enriched for TCM and TSCM phenotypes has been recently developed (159) in which, on average, 50% of the T cells were TCM and 46% TSCM. The authors reported that the results were consistently achieved even with very few T cells available to start. The use of CAR T cells enriched for TCM and TSCM has reached the clinic even in solid tumors: the GD2-CD28-OX40 CAR manufactured in IL-7/IL-15 (157) is currently in a phase I trial to treat patients with sarcoma, osteosarcoma, neuroblastoma, and melanoma (NCT02107963).

### Tissue Resident Memory Cells

Another memory T cell subset that may be of special importance, especially in treating solid tumors, are tissue resident memory cells (Trms), reviewed in (160). Trms have been shown to permanently reside at sites of prior infection or inflammation and quickly respond to pre-recognized antigen, recruiting other immune cells, and increasing the local anti-tumor immune response at a very early stage (161). CD8^+^ Trms are characterized by high surface levels of CD103 and the activation marker CD69 and low CD62L and CCR7, and it is believed that TGFβ and IL-15 are both important soluble factors that promote T cell differentiation to a Trm phenotype. Despite having many memory markers, Trms secrete high levels of cytokines such as granzyme B and perforin. Interestingly, Chang et al. (153) and Wakim et al. (162) found that Trms do not tend to have high T-bet as other memory T cells do. Mackay et al. (163) describe downregulation of T-bet, but with necessary residual activity, as one of the factors driving skin Trms, in addition to downregulation of Eomes.

While a significant amount of this research has been done on Trms in skin, some data shows that analysis of Trms across various tissues obtained from humans retain similar phenotypes, particularly in CD8^+^ Trms (164). One examination of the transcriptional profiles of Trms in multiple tissue types showed that the gene Hobit (“homolog of Blimp1 in T cells”) was upregulated, and together with Blimp1 was a driver of a Trm phenotype (165). CD103 was not expressed on all Trms described, which has also been shown in other tissues including the brain (162). Interestingly, the genes Hobit and Blimp1 were also upregulated in activated NK cells, suggesting a similar signaling pathway during activation of both Trms and NK cells. Other studies offer support for this parallel, including one by Lotem et al. (166) that reported regulation of activation and proliferation in both mature CD8^+^ T cells and NK cells by the transcription factor Runx3. High levels of Runx3 have been shown by several studies to decrease CD4^+^ and increase CD103 expression on T cells, biasing them toward a cytotoxic CD8^+^ Trm phenotype. In a study by Cruz-Guilloty et al. (167), Runx3 also was reported to drive an increase in Eomes expression and granzyme B and perforin secretion in differentiating CD8^+^ T cells, while T-bet expression peaked early, at around 2 days, and then decreased over a week of differentiation. The authors also reported Runx3 to regulate CD103 in resting NK cells. While ongoing research is required to parse out more information on Trms, induction of a Trm phenotype using TGFβ and IL-15, or via a genetic engineering, could be a powerful way to improve upon ACT or CAR T cell efficacy. In a study of TILs in lung cancer patients, transcriptome analysis showed that CD103 and other Trm-linked markers were significantly increased in the patients with high numbers of TILs; moreover, having a higher percentage of Trms was reported to predict better survival (168). In a study using an orthotopic head and neck cancer model in mice, a cancer vaccine was more successful after induction of Trms, and Trms were still detectable at the tumor site 90 days later (169). Additionally, Trms protected against tumor re-grafting even when recruitment of additional effector T cells was blocked, showing that Trms alone can mount a successful antitumor response and tumor rejection upon rechallenge. These data show that Trms may be critical to successful tumor infiltration and protection against tumor relapse, and the induction of a Trm phenotype is likely to increase therapeutic outcomes.

### Overcoming Other Immunosuppressive Factors in the TME

Administration of cytokines to polarize the tumor mileu to be more hospitable to T cells and improve CAR T cell recruitment and functionality has been tested in both preclinical and clinical trials. Local delivery of IL-12, which induces inflammatory immune cell recruitment, augmented the anti-tumor activity of adoptively transferred anti-VEGFR-2 CAR T cells and led to prolonged survival of mice bearing five different subcutaneous tumor types (170). In the study, treatment of IL-12 plus VEGFR2 CAR T cells, but neither alone, reduced VEGFR2-positive intratumoral MDSCs, providing strong support for the combination of IL-12 with CAR T cells. Due to positive responses like these, CAR T cells that constitutively secrete cytokines, termed “armored” CARs [reviewed by Yeku et al. (171)] have been created to enhance T cell infiltration and function in solid tumors (Figure 1). Particularly, the cytokine IL-12 has been an attractive tool for this. In a mouse xenograft model of ovarian cancer, MUC16 CAR/IL-12 T cells lengthened survival and showed increased persistence and tumor cytotoxicity (53). More recently, in a syngeneic mouse model of peritoneal carcinomatosis (metastasized from ovarian cancer), IP-delivery of MUC16 CAR/IL-12 T cells was found to confer longer survival, even when administered to mice with significant disease progression (67).

Some other strategies to boost CAR T cell function in the TME include inhibiting suppressive soluble factors, like adenosine, IDO1, and VEGF, and protecting against the immune suppression of non-tumor cells in the TME like MDSCs, TAMs, and stromal cells. In a study using HER2 CAR T cells in a syngeneic tumor model, blockade of the adenosine 2A receptor significantly improved the efficacy of the CAR T cells by enhancing activation and cytokine production (118). Additionally, the authors reported that PD-1 blockade further augmented the T cell immune response. Another study demonstrated significant slowing of tumor growth in a xenograft colon cancer model by combining blockade of IDO1 (negatively correlated with patient survival in colon cancer) with EGFRvIII CAR T cell transfer (172). VEGF blockade has been successful in solid tumors such as melanoma, and VEGF-targeted CARs have shown efficacy in multiple preclinical solid tumor models (131, 132, 170).

Increasing antitumor response can also involve either depleting anti-inflammatory cells in the TME or inducing more inflammatory phenotypes in other immune cells. Research in mouse breast cancer models has suggested that targeting TAMs may be effective for treating progressive cancer, as TAMs were associated with more anti-inflammatory activity and tumor immune evasion (173). Another study demonstrated that in murine ovarian cancer models, macrophages were associated with resistance to VEGF blockade. When macrophages were depleted, survival was prolonged, and in macrophage deficient mice, resistance was not observed unless macrophages were reintroduced into the tumors (174). TAMs are, therefore, a highly active subset of immune cells that seem to promote tumor survival and immune evasion. In a subcutaneous mouse model of ovarian cancer, tumor rejection by HER2 CAR T cells was shown to require the presence of M1 (inflammatory) macrophages and IFNγ receptors on stromal cells, demonstrating that tumor-specific attack by T cells, even functional ones, may not be enough to clear tumors; stromal cell targeting (for example, with FAP CARs) and recruitment of other types of inflammatory immune cells may be necessary (175). Depleting MDSCs can also improve T cell responses, as shown in a study with a GD2 CAR in which the CAR T cell alone had no anti-tumor activity in a xenograft sarcoma model, but in combination with MDSC reduction using all-trans retinoic acid, led to significant antitumor activity (176). Noman et al. (177) demonstrated *in vivo* that hypoxia in the TME plays a significant role in upregulation of PDL-1 on MDSCs and on their subsequent suppression of TILs. PDL-1 upregulation was determined to be dependent on HIF1α, and PDL-1 blockade prevented T cell suppression by MDSCs. In another study (described in section Tumor Infiltration) using a CEA CAR, blockade of PD-L1 positive MDSCs and Tregs in the TME augmented CAR T cell anti-tumor function (108).

### Intracellular Signaling Pathways of the CAR

It is also important to study the signaling pathway of the CAR itself, particularly how different costimulatory domains may affect T cell activation, metabolic needs, differentiation pathways, and the propensity to exhaust. Adding a costimulatory molecule to the original CD3ζ cytoplasmic domain revolutionized the functionality of the CAR T cell; now, there is a broad array of signaling molecules that can be used. The most common costimulatory molecules are 4-1BB and CD28, and depending on the CAR and tumor type, many studies focus on one or the other. Some have studied adding a third costimulatory domain, like ICOS or OX40. Some studies have demonstrated little significant advantage of one design over another. In a study comparing 4-1BB vs. CD28 in an EphA2 CAR, both CARs displayed equally potent antitumor activity in a xenograft mouse glioma model, and creating a third-generation CAR with both domains did not improve T cell performance over the second-generation CARs (27). In the study described in section Tumor Infiltration that used mesothelin/IL-7/CCL19 CARs to treat murine mesothelin-expressing PDA, there was also no difference between 4-1BBζ and CD28ζ CARs (66).

Many studies implicate 4-1BB as promoting superior differentiation phenotype and persistence. A recent study used phosphoproteomics to report on the kinetics of the 4-1BB vs. CD28 domains in CAR T cells. The authors found that 4-1BBζ CARs and CD28ζ CARs signaled through the same intermediates, but CD28ζ CARs had more and faster changes in protein phosphorylation, which seemed to drive them toward an effector phenotype. On the other hand, 4-1BBζ CARs were shown to express more memory-related genes and performed better *in vivo* than their CD28ζ CAR counterparts (178). Another study comparing 4-1BB and CD28 signaling in a PSCA CAR to treat patient derived prostate cancer xenografts found 4-1BB to be superior to CD28, with 4-1BBζ CARs leading to less exhaustion and better antigen selectivity (however, *in vitro* killing was equal between the two CARs) (179). In the aforementioned study of a regionally delivered HER2 CAR in xenograft models of brain-metastasized breast cancer, 4-1BBζ CARs also showed superior proliferation and less exhaustion than CD28ζ CARs (106). The evidence for 4-1BB preferentially expressing memory markers so far has been borne out clinically: an *ex vivo* study showed that in both CD19 and mesothelin CARs across multiple donors' T cells, 4-1BB promoted better proliferation, central memory differentiation, and greater levels of fatty acid oxidation and mitochondria generation than CD28, while CD28 was linked to increased glycolysis and an effector phenotype (6). Other 4-1BB based CARs from *in vivo* studies described in this review include HER2 CARs in models of medulloblastoma and gastric cancer (92,30); GD2 CARs in models of neuroblastoma and patients with melanoma (29, 30); mesothelin CARs in preclinical models of mesothelioma (64); and FAP CARs used in models of tumor associated stroma (102). Clinically, data is rare so far for solid tumors, but a case study described by Brown et al. (42) showed tumor regression induced by T cells expressing a 4-1BBζ IL13Rα2 CAR in a patient with glioblastoma.

Numerous studies have engineered effective CARs that signal through the CD28ζ domain, many of which target the same antigens and are used in similar disease models as 4-1BBζ-signaling CARs. These include humanized HER2 CARs that were shown to have a central memory phenotype in the context of treating breast cancer xenografts (35); IL13Rα2 CARs that showed proliferation and cytotoxicity in a mouse model of glioblastoma (41); and FAP CARs in IP mouse models of MPM (104). Other CARs mentioned in this review that use the CD28ζ costimulatory domain include L1CAM CARs for ovarian cancer in mice (45), MET CARs for MPM (48), MUC16 CARs in mouse models of ovarian cancer and peritoneal carcinomatosis (53, 67), and NKG2D CARs in Ewing's sarcoma models (54). Studies have also used CD28 with dual CARs, such as HER2/MUC1 bispecific CARs in *in-vitro* breast cancer models and HER2/IL13Rα2 CARs in xenograft glioma models (86, 87). Clinically, a HER2/CD28ζ CAR was used to treat progressive glioblastoma in a phase I trial that showed efficacy in some patients (38).

Finally, third generation CARs have also been studied in preclinical and clinical settings. A recent study comparing third generation GD2 CARs to treat *in vivo* models of neuroblastoma found 4-1BB/CD28 CARs to be superior to CD28/OX40ζ CARs in terms of activation, exhaustion, and *in vivo* antitumor efficacy (180). Successful *in vivo* studies using 4-1BB/CD28 third generation CARs include an ICAM-1 CAR for a mouse model of thyroid cancer (40), a GPC3 CAR in a patient derived xenograft of model of HCC (32), and a VEGFR2 CAR against multiple tumor types *in vivo* (161). However, the study that observed fatal toxicity with the use of a FAP CAR in syngeneic mice used a 4-1BB/CD28ζ third generation CAR, and the case report (mentioned in section 1) of a patient death was after administration of HER2 4-1BB/CD28ζ CAR T cells (58). A third generation mesothelin CAR using ICOS/4-1BBζ showed significantly better tumor control and better T cell persistence than ICOSζ or 4-1BBζ CARs alone in a mesothelin-expressing pancreatic xenograft NSG mouse model (181). This study also provided significant insight into the signaling pathways that may be required for optimal CAR T cell activation and differentiation. Lower surface CAR expression corresponded to less tonic signaling (signaling in the absence of antigen), which is linked to exhaustion and has been observed in both CD28ζ and 4-1BBζ CARs. Additionally, the authors found that ICOS/4-1BBζ CARs only performed better than second generation CARs when ICOS was proximal to the transmembrane domain. In the clinic, GD2 CARs with CD28/OX40 costimulatory domains are currently in phase I trials for neuroblastoma (31).

The site of gene integration has also been shown to have significant impact on CAR function. A study with a CD19/28ζ CAR used CRISPR to insert the CAR under the control of the TCR promoter (at the TRAC locus), while simultaneously knocking out the TCR via insertion of the CAR gene (182). The results indicated enhanced proliferation, more memory cells, and much less exhaustion. The TRAC locus CAR was also hypothesized to have reduced tonic signaling that would push T cells toward terminal differentiation and exhaustion.

### Future Directions and Conclusions

CAR T cell therapy remains extremely expensive, and patients with severely depleted immune systems may not have viable T cells for autologous CAR T cell generation; additionally, concerns about immunogenicity of certain CAR designs may render therapy less effective in patients that develop an immune response to the CAR. New approaches are needed to make CAR T cells not only functional, but also more efficient and accessible.

Technology such as CRISPR/Cas9 as a highly specific and efficient method of genome editing has become translatable to patients in the past few years. Beyond its use in generating IR knockout CARs, CRISPR/Cas9 has been used to knock out or replace the native TCR in CAR and TCR engineered T cells, which has been shown to provide higher antigen sensitivity and specificity (183). In addition to its implications for T cell function, the use of CRISPR is extremely promising in the field of CAR T cell therapy because it can be used to knock out HLA as well as the endogenous TCR, meaning CARs can be made from allogeneic cells without the threat of cross reactivity and GVHD or rejection. This could dramatically reduce the cost, time and resources required to generate CAR T cells for every patient (184). This has recently been done successfully with a TCR and HLA class I double knockout CAR19 (185). Investigators also developed a CAR that knocked out Fas as well as the TCR and HLA-1 genes, which showed enhanced antitumor activity *in vivo* against a leukemic cell line, with longer survival than unmodified CAR T cells. Thus, CRISPR can be used not only to knock out inhibitory receptors, but also to knock out the TCR and HLA to generate universal or “off the shelf” CARs (186). Moreover, these modifications can be accomplished simultaneously with high precision and efficiency.

Universal CARs have also been developed using other systems of genome editing, including transcription activator-like effector nucleases (TALENs), which create double-stranded breaks in DNA for efficient gene knockouts. Recently, TALEN-mediated editing was used to knock out the TCR-α chain in CAR19 T cells. The subsequent universal CAR T cells, which were from allogeneic donors, induced remission in two infants with B cell ALL (187). Before the advent of CRISPR/Cas9, zinc finger nucleases (ZFNs), proteins that recognize three base pairs at a time to bind to DNA, were also used to remove surface expression of molecules like HLA from allogeneic T cells (185, 188). (189) used ZFNs to disrupt both the TCR β- and α-chain genes while also lentivirally transducing the T cells to express a WT-1 recognizing TCR. This led to superior *in vivo* antitumor activity and eliminated off-target reactivity.

In some cases where the scFv is murine-derived, there is the potential for the development of anti-mouse antibodies that could reject the CAR T cells. Many studies have adopted the use of a humanized scFv, and these humanized CARs have also gone to clinical trials (8). However, humanizing the scFv is a long and onerous process and few fully humanized sequences are currently known. Thus, some recent studies have proposed alternatives to the scFv antigen-recognition domain. One of these alternatives is an affinity molecule from the type III domain of human fibronectin (Fn3), which is similar to the scFv of an antibody but is smaller and has a less complex structure without disulfide bonds, enabling easier *in vitro* generation of specific binding domains (190). Additionally, its smaller size may enable the Fn3 to recognize epitopes that scFvs cannot. Fn3 domains specific for CD20, CEA, EGFR, IGF-1R, and VEGFR2 have been developed. A VEGFR2-specific Fn3 CAR with a CD28ζ costimulatory domain showed *in vitro* antigen-specific T cell activation and cytotoxicity, and another Fn3 CAR engineered to target EGFR with both CD28 and 4-1BBζ costimulatory domains showed efficacy that was comparable to a traditional CAR against a xenograft lung cancer model (191, 192). Another alternative to the scFv is the use of antibody mimetic proteins, such as designed ankyrin repeat proteins (DARPins), synthetic proteins mimicking naturally occurring ankyrin membrane proteins that can be generated with antigen-binding specificity and are smaller and more stable than scFvs. Recently, a HER2-specific DARPin CAR was shown to perform as well as a traditional HER2 CAR *in vivo* against a human ovarian cancer cell line (193).

Significant research has been done with CAR T cells in terms of identifying target antigens, avoiding toxicity, improving CAR T cell trafficking and entry into the tumor site, and promoting better signaling, less exhaustion, and memory phenotypes in solid tumors. Additionally, combination therapy with checkpoint inhibitors, armored CARs, and suppression of other inhibitory factors in the TME has been shown to aid in CAR T cell efficacy in solid tumors, with some of these approaches already being used in clinical trials. Solid tumors pose a wide array of challenges that hematological malignancies do not, hence the need for multi-pronged strategies in addressing them. However, it is clear that our understanding of the TME is increasing at a rapid rate. As the signaling pathways between T cells and other TME cellular components, as well as the intracellular signaling cascades specific to CAR T cell activation and exhaustion, become further understood, CARs hold the promise for greater success in treating solid tumors.

## Author Contributions

MM did the primary research and wrote the manuscript. EM oversaw the preparation of the manuscript and edited the final draft.

### Conflict of Interest Statement

The authors declare that the research was conducted in the absence of any commercial or financial relationships that could be construed as a potential conflict of interest.
